# Structure simulation into a lamellar supramolecular network and calculation of the metal ions/ligands ratio

**DOI:** 10.1186/1752-153X-6-91

**Published:** 2012-08-29

**Authors:** Aurelia Visa, Maria Mracec, Bianca Maranescu, Valentin Maranescu, Gheorghe Ilia, Adriana Popa, Mircea Mracec

**Affiliations:** 1 Institute of Chemistry Timisoara of the Romanian Academy, 24 Mihai Viteazul Ave, Timisoara, RO-300223, Romania; 2 Faculty of Electronics and Telecommunications, University “Politehnica” of Timişoara, 2 Pârvan Ave, Timişoara, RO-300223, Romania; 3 MOLECULAR FORECAST Research Center, 125 Prof. Dr. Aurel Păunescu-Podeanu str, Timişoara, A 4, RO-300569, Romania

**Keywords:** Metal ions/ligands ratio, Metal organic frameworks, Lamellar structures, Supramolecular O_h_ complexes

## Abstract

**Background:**

Research interest in phosphonates metal organic frameworks (MOF) has increased extremely in the last two decades, because of theirs fascinating and complex topology and structural flexibility. In this paper we present a mathematical model for ligand/metal ion ratio of an octahedral (O_h_) network of cobalt vinylphosphonate (Co(vP)·H_2_O).

**Results:**

A recurrent relationship of the ratio between the number of ligands and the number of metal ions in a lamellar octahedral (O_h_) network Co(vP)·H_2_O, has been deducted by building the 3D network step by step using HyperChem 7.52 package. The mathematical relationship has been validated using X ray analysis, experimental thermogravimetric and elemental analysis data.

**Conclusions:**

Based on deducted recurrence relationship, we can conclude prior to perform X ray analysis, that in the case of a thermogravimetric analysis pointing a ratio between the number of metal ions and ligands number around 1, the 3D network will have a central metal ion that corresponds to a single ligand. This relation is valid for every type of supramolecular network with divalent metal central ion O_h_ coordinated and bring valuable information with low effort and cost.

## Background

Metal-organic frameworks have found various applications [1-5]. In recent years, considerable interest has been developed in the use of complexing molecules containing phosphonate moieties [6-8]. These materials have potential applications as ion exchangers, sensors, in catalysis and in magnetism [9-12]. Metal organic frameworks are predictable to outline the starting point of future molecular machinery design. Most networks are based on metal organic carboxylic derivatives that form a controlled supramolecular structure with transition metal ions. Phosphonates metal organic frameworks are quickly gaining a central position amongst the various families of MOF materials. There are a broad variety of phosphonate ligands that can be used, containing aliphatic or aromatic connectivity and functional groups [13-15]. They display a number of similarities, but also diversities to the carboxylates. Phosphonate building blocks hold three oxygen atoms linked to the phosphorus atom in the coordinating moiety, compared to two oxygen atoms for carboxylates. This increases the potential to obtain new structures.

Phosphonate metal organic framework materials synthesis can be perform in various ways leading to products with similar or different supramolecular structures. The method leads to a combinatorial approach. Phosphonic acid derivatives make ideal candidates as spacers for metal-organic frameworks which can incorporate virtually any organic group. Phosphonic acids in combination with a metal precursor let phosphonates MOF products to have a layered supramolecular structure in which the metal centers are bridged by the phosphonate group, revealing a variety of 1D chain, 2D layer, and 3D network with micropores. D.A. Knight reports the molecular layered structure of copper vinylphosphonate and cadmium vinylphosphonate [16,17]. The cadmium ion is six-coordinate in a distorted octahedral (O_h_) environment. Five of the coordinated oxygen atoms are provided by the phosphate group and the sixth originate from a bound water molecule.

## Results and discussion

Cobalt vinylphosphonate was obtained in our labs by the reaction of Co(NO_3_)_2_·6H_2_O, vinyl phosphonic acid (vP) in equimolecular ratio and hydrothermal conditions, having similar coordination geometry with cadmium vinylphosphonate, nikel vinylphosphonate, zinc vinylphosphonate and cobalt phenylvinylphosphonate [18].

In this paper we have chosen the cobalt vinylphosphonate for study because of his semiconducting properties. The details about this special properties as well as the geometric properties (bond lengths, bond angles, torsion angles) and electronic properties (energetic levels, charges, heats of formation, ZPE, ν_min_, ν_max_ ) from semi-empirical PM3 calculation data are presented in our previous study [19].

Using the HyperChem 7.52 package, we have built the 3D network using a step by step approach in two ways: *on row* and *on column*.

Complexes with up to 8 Co^2+^ ions on the row are rendered in Table 1 which shows the ions and ligands numbers of all combinations up to an 8x8 network, *k* representing the number of rows and *n* the number of columns.

**Table 1:** **The number of Co**^**2+**^**ions and the number of vP ligands resulted by building step by step an 8x8 network**

** *k* **	** *n* **	**1**	**2**	**3**	**4**	**5**	**6**	**7**	**8**
1	Co^2+^	1	2	3	4	5	6	7	8
	vP	4	6	8	10	12	14	16	18
2	Co^2+^	2	4	6	8	10	12	14	16
	vP	7	10	13	16	19	22	25	28
3	Co^2+^	3	6	9	12	15	18	21	24
	vP	10	14	18	22	26	30	34	38
4	Co^2+^	4	8	12	16	20	24	28	32
	vP	13	18	23	28	33	38	43	48
5	Co^2+^	5	10	15	20	25	30	35	40
	vP	16	22	28	34	40	46	52	58
6	Co^2+^	6	12	18	24	30	36	42	48
	vP	19	26	33	40	47	54	61	62
7	Co^2+^	7	14	21	28	35	42	49	56
	vP	22	30	38	46	54	62	70	78
8	Co^2+^	8	16	24	32	40	48	56	64
	vP	25	34	43	52	61	70	79	88

To demonstrate experimental observation, Table 1 values can be rewritten as presented in Table 2. Into this new arrangement it can be remarked that there is a certain regularity meaning that a recurrent relationship can be extracted. By expressing the Table 2 values regarding corresponding row and column results in Table 3.

**Table 2:** **Rearrangement of the data shown in Table**1

** *k* **	** *n* **	**1**	**2**	**3**	**4**	**5**	**6**	**7**	**8**
1	Co^2+^	1 = 1·1	2 = 1·2	3 = 1·3	4 = 1·4	5 = 1·5	6 = 1·6	7 = 1·7	8 = 1·8
	vP	4 = 2·2 + 0	6 = 2·3 + 0	8 = 2·4 + 0	10 = 2·5 + 0	12 = 2·6 + 0	14 = 2·7 + 0	16 = 2·8 + 0	18 = 2·9 + 0
2	Co^2+^	2 = 2·1	4 = 2·2	6 = 2·3	8 = 2·4	10 = 2·5	12 = 2·6	14 = 2·7	16 = 2·8
	vP	7 = 3·2 + 1	10 = 3·3 + 1	13 = 3·4 + 1	16 = 3·5 + 1	19 = 3·6 + 1	22 = 3·7 + 1	25 = 3·8 + 1	28 = 3·9 + 1
3	Co^2+^	3 = 3·1	6 = 3·2	9 = 3·3	12 = 3·4	15 = 3·5	18 = 3·6	21 = 3·7	24 = 3·8
	vP	10 = 4·2 + 2	14 = 4·3 + 2	18 = 4·4 + 2	22 = 4·5 + 2	26 = 4·6 + 2	30 = 4·7 + 2	34 = 4·8 + 2	38 = 4·9 + 2
4	Co^2+^	4 = 4·1	8 = 4·2	12 = 4·3	16 = 4·4	20 = 4·5	24 = 4·6	28 = 4·7	32 = 4·8
	vP	13 = 5·2 + 3	18 = 5·3 + 3	23 = 5·4 + 3	28 = 5·5 + 3	33 = 5·6 + 3	38 = 5·7 + 3	43 = 5·8 + 3	48 = 5·9 + 3
5	Co^2+^	5 = 5·1	10 = 5·2	15 = 5·3	20 = 5·4	25 = 5·5	30 = 5·6	35 = 5·7	40 = 5·8
	vP	16 = 6·2 + 4	22 = 6·3 + 4	28 = 6·4 + 4	34 = 6·5 + 4	40 = 6·6 + 4	46 = 6·7 + 4	52 = 6·8 + 4	58 = 6·9 + 4
6	Co^2+^	6 = 6·1	12 = 6·2	18 = 6·3	24 = 6·4	30 = 6·5	36 = 6·6	42 = 6·7	48 = 6·8
	vP	19 = 7·2 + 5	26 = 7·3 + 5	33 = 7·4 + 5	40 = 7·5 + 5	47 = 7·6 + 5	54 = 7·7 + 5	61 = 7·8 + 5	62 = 7·9 + 5
7	Co^2+^	7 = 7·1	14 = 7·2	21 = 7·3	28 = 7·4	35 = 7·5	42 = 7·6	49 = 7·7	56 = 7·8
	vP	22 = 8·2 + 6	30 = 8·3 + 6	38 = 8·4 + 6	46 = 8·5 + 6	54 = 8·6 + 6	62 = 8·7 + 6	70 = 8·8 + 6	78 = 8·9 + 6
8	Co^2+^	8 = 8·1	16 = 8·2	24 = 8·3	32 = 8·4	40 = 8·5	48 = 8·6	56 = 8·7	64 = 8·8
	vP	25 = 9·2 + 7	34 = 9·3 + 7	43 = 9·4 + 7	52 = 9·5 + 7	61 = 9·6 + 7	70 = 9·7 + 7	79 = 9·8 + 7	88 = 9·9 + 7

**Table 3:** **A rewrite of the values from Table 2 versus *n* and *k,* in order to build a recurrent relationship**

** *k* **	** *n* **	**1**	**2**	**3**	**4**	**5**	**6**	**7**	**8**
1	Co^2+^	1 = 1·1	2 = 1·2	3 = 1·3	4 = 1·4	5 = 1·5	6 = 1·6	7 = 1·7	8 = 1·8
	vP	4 = (1 + 1)·(1 + 1) + (1–1)	6 = (1 + 1)·(2 + 1) + (1–1)	8 = (1 + 1)·(3 + 1) + (1–1)	10 = (1 + 1)·(4 + 1) + (1–1)	12 = (1 + 1)·(5 + 1) + (1–1)	14 = (1 + 1)·(6 + 1) + (1–1)	16 = (1 + 1)·(7 + 1) + (1–1)	18 = (1 + 1)·(8 + 1) + (1–1)
2	Co^2+^	2 = 1·2	4 = 2·2	6 = 2·3	8 = 2·4	10 = 2·5	12 = 2·6	14 = 2·7	16 = 2·8
	vP	7 = (2 + 1)·(1 + 1) + (2–1)	10 = (2 + 1)·(2 + 1) + (2–1)	13 = (2 + 1)·(3 + 1) + (2–1)	16 = (2 + 1)·(4 + 1) + (2–1)	19 = (2 + 1)·(5 + 1) + (2–1)	22 = (2 + 1)·(6 + 1) + (2–1)	25 = (2 + 1)·(7 + 1) + (2–1)	28 = (2 + 1)·(8 + 1) + (2–1)
3	Co^2+^	3 = 3·1	6 = 3·2	9 = 3·3	12 = 3·4	15 = 3·5	18 = 3·6	21 = 3·7	24 = 3·8
	vP	10 = (3 + 1)·(1 + 1) + (3–1)	14 = (3 + 1)·(2 + 1) + (3–1)	18 = (3 + 1)·(3 + 1) + (3–1)	22 = (3 + 1)·(4 + 1) + (3–1)	26 = (3 + 1)·(5 + 1) + (3–1)	30 = (3 + 1)·(6 + 1) + (3–1)	34 = (3 + 1)·(7 + 1) + (3–1)	38 = (3 + 1)·(8 + 1) + (3–1)
4	Co^2+^	4 = 4·1	8 = 4·2	12 = 4·3	16 = 4·4	20 = 4·5	24 = 4·6	28 = 4·7	32 = 4·8
	vP	13 = (4 + 1)·(1 + 1) + (4–1)	18 = (4 + 1)·(2 + 1) + (4–1)	23 = (4 + 1)·(3 + 1) + (4–1)	28 = (4 + 1)·(4 + 1) + (4–1)	33 = (4 + 1)·(5 + 1) + (4–1)	38 = (4 + 1)·(6 + 1) + (4–1)	43 = (4 + 1)·(7 + 1) + (4–1)	48 = (4 + 1)·(8 + 1) + (4–1)
5	Co^2+^	5 = 5·1	10 = 5·2	15 = 5·3	20 = 5·4	25 = 5·5	30 = 5·6	35 = 5·7	40 = 5·8
	vP	16 = (5 + 1)·(1 + 1) + (5–1)	22 = (5 + 1)·(2 + 1) + (5–1)	28 = (5 + 1)·(3 + 1) + (5–1)	34 = (5 + 1)·(4 + 1) + (5–1)	40 = (5 + 1)·(5 + 1) + (5–1)	46 = (5 + 1)·(6 + 1) + (5–1)	52 = (5 + 1)·(7 + 1) + (5–1)	58 = (5 + 1)·(8 + 1) + (5–1)
6	Co^2+^	6 = 6·1	12 = 6·2	18 = 6·3	24 = 6·4	30 = 6·5	36 = 6·6	42 = 6·7	48 = 6·8
	vP	19 = (6 + 1)·(1 + 1) + (6–1)	26 = (6 + 1)·(2 + 1) + (6–1)	33 = (6 + 1)·(3 + 1) + (6–1)	40 = (6 + 1)·(4 + 1) + (6–1)	47 = (6 + 1)·(5 + 1) + (6–1)	54==(6 + 1)·(6 + 1) + (6–1)	61 = (6 + 1)·(7 + 1) + (6–1)	62 = (6 + 1)·(8 + 1) + (6–1)
7	Co^2+^	7 = 7·1	14 = 7·2	21 = 7·3	28 = 7·4	35 = 7·5	42 = 7·6	49 = 7·7	56 = 7·8
	vP	22 = (7 + 1)·(1 + 1) + (7–1)	30 = (7 + 1)·(2 + 1) + (7–1)	38 = (7 + 1)·(3 + 1) + (7–1)	46 = (7 + 1)·(4 + 1) + (7–1)	54 = (7 + 1)·(5 + 1) + (7–1)	62 = (7 + 1)·(6 + 1) + (7–1)	70 = (7 + 1)·(7 + 1) + (7–1)	78 = (7 + 1)·(8 + 1) + (7–1)
8	Co^2+^	8 = 8·1	16 = 8·2	24 = 8·4	32 = 8·4	40 = 8·5	48 = 8·6	56 = 8·7	64 = 8·8
	vP	25 = (8 + 1)·(1 + 1) + (8–1)	34 = (8 + 1)·(2 + 1) + (8–1)	43 = (8 + 1)·(3 + 1) + (8–1)	52 = (8 + 1)·(4 + 1) + (8–1)	61 = (8 + 1)·(5 + 1) + (8–1)	70 = (8 + 1)·(7 + 1) + (8–1)	79 = (8 + 1)·(7 + 1) + (8–1)	88 = (8 + 1)·(8 + 1) + (8–1)

Table 3 offer an overview for the value expressing the total number of ligands for a given complex versus row number *k* and column number *n*, leading to the recurrent relationship $\left(k+1\right)\left(n+1\right)+\left(k-1\right)$which represent a series.

If we note the number of cobalt metal ions from cobalt vinylphosphonate with *n·k* and the number of vP ligands with $\left(k+1\right)\left(n+1\right)+\left(k-1\right)$, where *k* = 1,2,3,… N, then the ratio between the number of ligands and metal ions is given by the following equivalent relation (1):

(1) $$\frac{\left(n+1\right)\left(k+1\right)+\left(k-1\right)}{nk}=\frac{nk\left(1+\frac{1}{n}\right)\left(1+\frac{1}{k}\right)+nk\left(\frac{1}{n}-\frac{1}{nk}\right)}{nk}=\left(1+\frac{1}{n}\right)\left(1+\frac{1}{k}\right)+\left(\frac{1}{n}-\frac{1}{nk}\right)$$

In the case of a supra-molecular monolayer network, we can consider that *n* and *k* are very high, and mathematically it can be considered that they go to infinity, leading to the equivalent relation (2):

(4) $$\lim_{\left(n\to \infty ;k\to \infty \right)}\left[\frac{\left(n+1\right)\left(k+1\right)+\left(k-1\right)}{nk}\right]=\lim_{\left(n\to \infty ;k\to \infty \right)}\left[\left(1+\frac{1}{n}\right)\left(1+\frac{1}{k}\right)+\left(\frac{1}{n}-\frac{1}{nk}\right)\right]=\left(1+0\right)\left(1+0\right)+0=1$$

By increasing the number of central metal ions, the ratio between the number of ligands and the number of ions in a lamellar octahedral (O_h_) network Co(vP)·H_2_O is converging to 1.

Therefore at the limit, for large *n* and *k*, each metal ion is correlated with a single ligand. This mathematical result can be checked as in the next example: if we assume that synthesis of 10^-3^ mol complex (1 mmol) result into 10^6^ crystals, will relate this to a mole of substance (6.23·10^23^ Co^2+^ ions, and vP, respectively), which means that in each crystal *n, k* is of the order of 6.23·10^23^/10^6^ = 6.23·10^14^, thus the number of rows and columns in a single crystal is very high and constitutes a supramolecular structure.

### Experimental

From elemental analysis results it has been observed that the calculated and found percentage for C, H and P are similar. [Co(C_2_H_3_PO_3_)^.^ H_2_O]_n;_ Anal. Calcd. for C_2_H_5_CoO_4_P: C, 13.13; H, 2.75; P, 16.93. Found: C, 13.1; H, 2.77; P, 16.89.

### Determination of phosphorus content

10 mL of distilled water was added in an Erlenmeyer flask with a spiraled platinum wire cork and the oxygen was bubbled in for 5 min. The 4–5 mg of the complex [Co(C_2_H_5_PO_3_)^.^H_2_O] weighed was wrapped in filter paper and was fixed in the platinum wire. Then, the sample was burned in an oxygen atmosphere, the flask being tightly closed. The sample was left in the closed flask for 30 min for the resulting gas (P_2_O_5_) to be absorbed in the water. Then, the spiraled platinum wire was rinsed with 20 mL distilled water. Next, 1 g of hexamethylenetetramine was added and the solution was boiled for 10 min. The solution obtained was titrated at 80 °C with an aqueous solution of cerium (III) 0.005 M in the presence of Eryochrome black T as indicator. The color was altered from blue to purple.

The phosphorus content in the sample is calculated with the relation (3):

(5) $$\%P=\frac{VCe^{\mathrm{III}}\cdot F\cdot 15.49}{m_{p}}$$

where: *V*_*Ce*_^*III*^ is the volume of solution of cerium (III) 0.005 M used to titration in mL, *F* is the factor of solution *Ce*^*III*^ (1.0309), 15.49 is a constant value and *m*_*p*_ is the weight of probe in mg.

The final phosphorus content was determined as an average value for the three determinations by described procedure [20].

The cobalt percentage in the sample is around the calculated weight percentage, having the value 32.2. Ratio between molar percentage of Co ion and phosphorus ligand is 1, confirmed by thermogravimetric analysis result.

## Conclusion

Starting from a particular case of a supramolecular structure [Co^II^(C_2_H_3_PO_3_)^.^ H_2_O]_n_ with Co^2+^ ion O_h_ coordinated, it was deducted a general recurrence relation of the ratio between the number of the Co central ions and vP ligands. It was demonstrated that for a supramolecular network with central ion O_h_ coordinated, for large numbers of columns *n* and rows *k* in the network, at the limit of the recurrence relationship, each metal ion correlates a single ligand.

This mathematical result was validated by X ray analysis, elemental analysis and thermogravimetric experimental data.

Using deducted relationship, we can conclude fast and with low cost, prior to perform X ray analysis, that in the case of a thermogravimetric analysis pointing a ratio between the number of metal ions and ligands number around 1, the 3D network will have a central metal ion which is coordinated octahedral with a coordinated water molecule and four bidentate (tridentate) ligands, bound by four neighbor metal ions.

## Method

According to Y. Chan, metal organic framework is expecting to form the basis of future molecular machinery design [21]. In order to find a recurrence relationship that can be calculated by the ratio between the number of ligands and the number of ions in a lamellar octahedral (O_h_) network Co(vP)·H_2_O, we have previously performed the X ray analysis of the structure and then using the HyperChem 7.52 package we have built the network step by step in both ways: *on row* and *on column*.

On row: first it is built a horizontal line linking a number of Co^2+^ ions complexed with vP between them and finally the network is built by linking identical rows.

On column: first it is built a vertical line linking Co^2+^ ions complexed with vP between them and finally the network is built by linking identical columns.

With both approaches a squared network 8 x 8 Co^2+^ ions will be built in order to deduct a recurrent relationship. It is obvious that the two ways of building will lead to a unique network, but the array of ligands on the line and on the column is not symmetrical with respect to the diagonal.

For preserving the neutrality of the complex molecule, marginal oxygen atoms, which are not linked to Co, will be linked to the hydrogen atoms shown in figures as *m*H, in order to facilitate the counting of the ligands.

**Figure 1:**
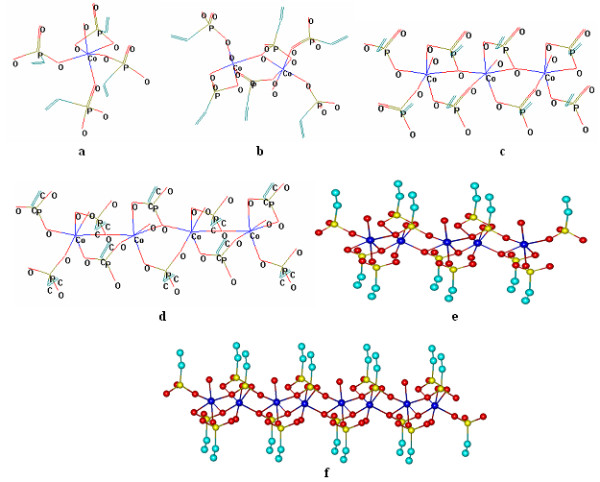
**3D network elements on a row: a) [Co(vP)**_**4**_**·3 H·H**_**2**_**O]; b) [Co**_**2**_**(vP)**_**6**_**·3 H·2H**_**2**_**O]; c) [Co**_**3**_**(vP)**_**8**_**·4 H·3H**_**2**_**O]; d)[Co**_**4**_**(vP)**_**10**_**·4 H·4H**_**2**_**O]; e) [Co**_**5**_**(vP)**_**12**_**·5 H·5H**_**2**_**O]; f)[Co**_**8**_**(vP)**_**18**_**·6 H·8H**_**2**_**O].**

First row of the network is built starting with a Co^2+^ ion and 4 vP ligands leading to the complex [Co(vP)_4_·3 H·H_2_O]. For two ions of Co^2+^, building the row lead to the complex [Co_2_(vP)_6_·3 H ·2H_2_O] which uses 6 vP ligands (Figure 1).

For 3 Co^2+^ ions in a row, the resulted complex [Co_3_(vP)_8_·4 H·3H_2_O] uses 8 vP ligands (Figure 1). For 4 Co^2+^ ions in a row, the complex [Co_4_(vP)_10_·4 H·4H_2_O] uses 10 vP ligands. For 5 Co^2+^ ions in a row the complex [Co_5_(vP)_12_·5 H·5H_2_O] uses 12 vP ligands. All row complexes [*1,6*]; [*1,7*]; [*1,8*] will be built in same manner.

Complexes with up to 8 Co^2+^ ions on the row are rendered in Table 1.

**Figure 2:**
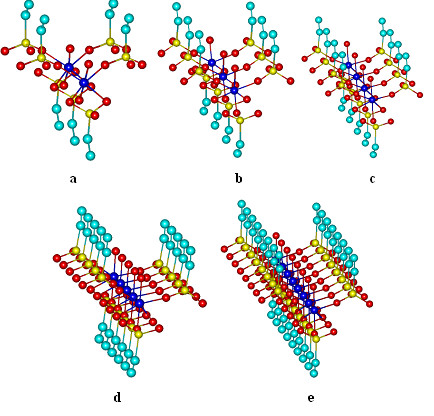
**3D network elements on a column : a) [Co**_**2**_**(vP)**_**7**_**·5 H·2H**_**2**_**O]; b) [Co**_**3**_**(vP)**_**10**_**·7 H·3H**_**2**_**O]; c) [Co**_**4**_**(vP)**_**13**_**·9 H·4H**_**2**_**O]; d) [Co**_**5**_**(vP)**_**16**_**·11 H·5H**_**2**_**O]; e) [Co**_**8**_**(vP)**_**25**_**·17 H·8H**_**2**_**O].**

To prove that the matrix containing the ligands is not symmetrical, we build in the same way the first column (Figure 2). Since the elements of diagonal belongs to the row and column at the same time, the item [*1,1*] was previously described (Figure 1). Similarly all complexes [*2,1*], [*3,1*], … [*8,1*], are constructed (Figure 2). The number of ligands, from 1 at 8 Co^2+^ ions on the first column is provided in Table 1.

From the presented values can be seen that the number of ligands for a column is not identical with the number of ligands for a line. If the geometries are compared from Figure 1 along with those in Figure 2, it can be noted that in a line water molecules are oriented alternatively: for odd Co^2+^ ions are facing upwards, and for even Co^2+^ ions are pointing down. On columns, water molecules are always oriented into the same part of the plane.

**Figure 3:**
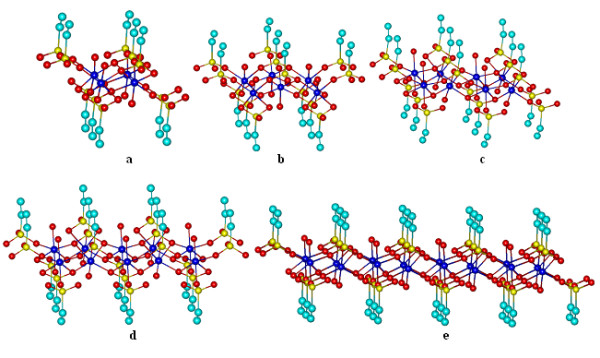
**3D network elements with two rows: a) [Co**_**4**_**(vP)**_**10**_**·5 H·4H**_**2**_**O]; b)[Co**_**6**_**(vP)**_**13**_**·5 H·6H**_**2**_**O]; c) [Co**_**8**_**(vP)**_**16**_**·6 H·6H**_**2**_**O]; d) [Co**_**10**_**(vP)**_**19**_**·7 H·10H**_**2**_**O]; …; e) [Co**_**16**_**(vP)**_**28**_**·8 H·16H**_**2**_**O)].**

With a row already built we can start the network assembly by adding step by step the first row to the second row, thus a complex with two rows and two columns [Co_4_(vP)_10_·5 H·4H_2_O] has 4 Co^2+^ and 10 vP ligands. Two rows and three columns [Co_6_(vP)_13_·5 H·6H_2_O] has 6 Co^2+^ ions 13 vP ligands. Two rows and four columns [Co_8_(vP)_16_·6 H·8H_2_O] has 8 Co^2+^ ions and 16 vP ligands. Two rows and five columns [Co_10_(vP)_19_·7 H·10H_2_O] has 10 Co^2+^ ions and 19 vP ligands (Figure 3).

In the same way the complexes [*2,6*], [*2,7*], [*2,8*] are constructed, and the number of ligands, up to 16 Co^2+^ ions in a network with two rows is rendered in Table 1.

**Figure 4:**
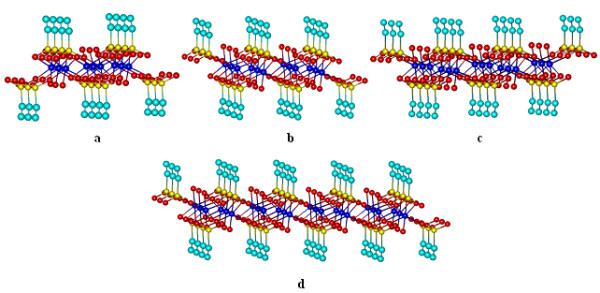
**3D network elements with three rows: a)[Co**_**9**_**(vP)**_**18**_**·7 H·9H**_**2**_**O]; b) [Co**_**12**_**(vP)**_**22**_**·8 H·12H**_**2**_**O]; c) [Co**_**15**_**(vP)**_**26**_**·9 H·15H**_**2**_**O]; d) [Co**_**24**_**(vP)**_**38**_**·10 H·24H**_**2**_**O)].**

For three rows, the network results as flows: three rows and two columns complex [Co_6_(vP)_14_·7 H·6H2O)] has 6 Co^2+^ ions and 14 vP ligands. Three rows and three columns complex [Co_9_(vP)_18_·7 H·9H_2_O] has 9 Co^2+^ ions and 18 vP ligands. Three rows and four columns complex [Co_12_(vP)_22_·8 H·12H_2_O] has 12 Co^2+^ ions and 22 vP ligands. Three rows and five columns complex [Co_15_(vP)_26_·9 H·15H_2_O] has 15 Co^2+^ ions and 26 vP ligands (Figure 4).

In the same way the complexes [*3,6*], [*3,7*], [*3,8*] are built and the number of ligands up to 24 Co^2+^ ions in a network with three rows are rendered in Table 1.

In the same way, networks with 4, 5, 6, 7 and 8 rows are built. In the construction of the complexes on the rows, first component [*1, k*], *k* = 2, …, 8 was not longer presented because it has been previously shown in Figure 2.

**Figure 5:**
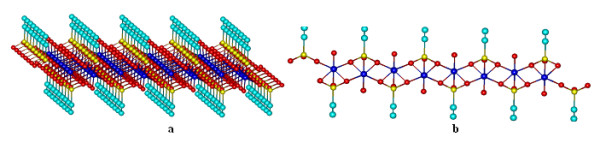
**3D network elements with eight rows and eight columns [Co**_**64**_**(vP)**_**88**_**·20 H·64H**_**2**_**O)] seen from the perspective and from the front.**

As in Figures 1–4, the structures were presented from the perspective; the images may be delusory and does not reveal clearly if all Co^2+^ ions are or not into the same plane. In Figure 5 a 8x8 network structure is presented seen from front, where it can be observed that Co^2+^ ions resides in two separate planes: Co^2+^ ions in odd columns reside in one plane, and those of even columns reside in a different plane. As can be noticed from Figures 1–4, ligands number for each complex can be deducted easily from vinyl groups (−CH = CH_2_) number. For this reason the 3D geometry of complexes were presented from the perspective.

Table 1 contains the total number of Co^2+^ ions and total number of vP ligands for a square 8 x 8 = 64 network of Co^2+^ions. At a first look it might be concluded that between the number of row elements and column elements there is no recurrent relationship, but the ratio between the number of ligands and the number of Co^2+^ ions decreases from 4 for element [*1,1*] at 1.375 for complex [*8,8*]. This fact leads us to search a recurrent relationship between the number of vP ligands and the number of Co^2+^ ions in a monolayer supramolecular network which should have a finite limit.

This limit is enforced by the experimental facts. In the synthesis of supramolecular structure, Co^2+^ salt and vP^2-^ were used in 1:1 molar ratio. Thermal analysis of the product has revealed that the ratio between the number of vP ligands and the number of ions Co^2+^ is 1. This means that for a large structure with infinite *n* and *k*, the ratio between number of ligands and number of central ions for an octahedral structure must be 1.

## Competing interests

The authors declare that they have no competing interest.

## Authors' contributions

AV, BM synthesized the compounds and prepared the manuscript, MM, MM contributed in structures design and discussion, AP, GI characterized the compounds, VM helped to draft the manuscript. All authors read and approved the final manuscript.
